# Explainable AI in Clinical Decision Support Systems: A Meta-Analysis of Methods, Applications, and Usability Challenges

**DOI:** 10.3390/healthcare13172154

**Published:** 2025-08-29

**Authors:** Qaiser Abbas, Woonyoung Jeong, Seung Won Lee

**Affiliations:** 1 Department of Electrical Engineering, Institute of Space Technology, Islamabad 44000, Pakistan; abbas72qaiser@gmail.com; 2 Department of Metabiohealth, Institute for Cross-Disciplinary Studies, Sungkyunkwan University, Suwon 16419, Republic of Korea; hbm06014@skku.edu; 3 Department of Precision Medicine, School of Medicine, Sungkyunkwan University, Suwon 16419, Republic of Korea; 4 Department of Artificial Intelligence, Sungkyunkwan University, Suwon 16419, Republic of Korea; 5 Personalized Cancer Immunotherapy Research Center, School of Medicine, Sungkyunkwan University, Suwon 16419, Republic of Korea; 6 Department of Family Medicine, Kangbuk Samsung Hospital, School of Medicine, Sungkyunkwan University, Seoul 03181, Republic of Korea

**Keywords:** explainable artificial intelligence (XAI), healthcare AI, human-centered AI, healthcare, medicine, clinical decision support systems (CDSSs)

## Abstract

**Background:** Theintegration of artificial intelligence (AI) into clinical decision support systems (CDSSs) has significantly enhanced diagnostic precision, risk stratification, and treatment planning. AI models remain a barrier to clinical adoption, emphasizing the critical role of explainable AI (XAI). **Methods:** This systematic meta-analysis synthesizes findings from 62 peer-reviewed studies published between 2018 and 2025, examining the use of XAI methods within CDSSs across various clinical domains, including radiology, oncology, neurology, and critical care. Model-agnostic techniques such as visualization models like Gradient-weighted Class Activation Mapping (Grad-CAM) and attention mechanisms dominated in imaging and sequential data tasks. **Results:** However, there are still gaps in user-friendly evaluation, methodological transparency, and ethical issues, as seen by the absence of research that evaluated explanation fidelity, clinician trust, or usability in real-world settings. In order to enable responsible AI implementation in healthcare, our analysis emphasizes the necessity of longitudinal clinical validation, participatory system design, and uniform interpretability measures. **Conclusions:** This review offers a thorough analysis of the state of XAI practices in CDSSs today, identifies methodological and practical issues, and suggests a path forward for AI solutions that are open, moral, and clinically relevant.

## 1. Introduction

AI has emerged as a transformative force in modern healthcare, particularly through its integration into clinical decision support systems (CDSSs) [1,2]. CDSSs are computational tools designed to assist clinicians in making data-driven decisions by providing evidence-based insights derived from patient data, medical literature, clinical guidelines, and real-time health analytics [3]. These systems aim to improve diagnostic accuracy, improve patient outcomes, and reduce medical errors [4]. With the advent of AI, particularly machine learning (ML) and deep learning (DL) techniques, CDSSs have become more powerful, capable of uncovering complex patterns in vast datasets and delivering predictive and prescriptive analytics with unprecedented speed and precision [5,6].

However, despite these advancements, a critical barrier to the widespread adoption of AI in healthcare is the lack of transparency and interpretability in model decision-making processes [7,8]. Many AI models, especially deep neural networks, operate as “black boxes,” as they provide predictions or classifications without offering clear explanations for their outputs [9]. In high-stakes domains such as medicine, in which clinicians must justify their decisions and ensure patient safety, this opacity is a significant drawback. Physicians are understandably reluctant to rely on recommendations from systems they do not fully understand, especially when these decisions impact patients’ lives. This has led to increasing demand for XAI, a subfield of AI that focuses on creating models with behavior and predictions that are understandable and trustworthy to human users [10,11].

Explainable AI aims to make AI systems more transparent, interpretable, and accountable. It encompasses a wide range of techniques, including model-agnostic methods like LIME (Local Interpret Model-agnostic Explanations) and SHAP (SHapley Additive exPlanations), as well as model-specific approaches such as decision trees, attention mechanisms, and saliency maps like Grad-CAM [12,13]. These methods are designed to provide insights into which features influence a model’s decision, how sensitive the model is to input variations, and how the trustworthiness of its predictions varies across contexts [14]. The goal is not only to satisfy regulatory and ethical requirements but also to foster human–AI collaboration by improving the understanding and confidence of clinicians in AI-driven tools [15,16].

The importance of XAI in healthcare cannot be overstated. Regulatory bodies such as the U.S. Food and Drug Administration (FDA) and the European Medicines Agency (EMA) are increasingly emphasizing the need for transparency and accountability in AI-based medical devices [17,18]. Explainability is also central to the ethical principles of AI, including fairness, accountability, and transparency (FAT) [19,20]. In clinical settings, explainability supports informed consent, shared decision making, and the ability to contest or audit algorithmic decisions. Furthermore, explainability can improve model debugging and development, helping researchers and engineers identify biases, data quality issues, and unintended outcomes [21].

CDSSs that incorporate XAI can provide multiple benefits. For example, in diagnostic imaging, XAI can highlight specific regions of interest on radiographs or MRIs that contribute to a diagnosis, allowing radiologists to verify and validate the model’s conclusions [22,23]. In predictive analytics, such as prediction of sepsis or risk of readmission to the ICU, XAI methods can identify key contributing factors such as vital signs, laboratory values, and patient history. This not only aids in clinical interpretation but also aligns AI recommendations with clinical reasoning, thereby increasing user trust and adoption [24,25].

Despite these advantages, implementating XAI in healthcare presents several challenges. One major issue is the trade-off between model accuracy and interpretability. Simpler models such as logistic regression and decision trees are easier to explain but may lack the predictive power of complex neural networks [26,27]. On the contrary, methods used to explain black-box models can introduce approximation errors or oversimplify prediction reasoning [28]. Another challenge is the lack of standardized metrics to evaluate the quality and usefulness of explanations. What is considered a good explanation depends on the clinical context, the user’s expertise, and the decision at hand [29,30].

Moreover, there is a need for user-centered design in the development of XAI systems. Clinicians have different needs and cognitive styles, and not all explanations are equally meaningful or useful for every user [31,32]. Effective XAI must be tailored to the target audience, be it clinicians, patients, regulators, or developers. This includes considerations of how explanations are presented (visual, textual, or interactive), the granularity of detail, and the timing of explanation delivery. Research on human–computer interaction (HCI) and cognitive psychology plays a vital role in informing these design choices [33].

Another consideration is the real-world integration of XAI into clinical workflows. Many studies on XAI in healthcare remain in the proof-of-concept stage or are only tested on retrospective datasets [34,35]. To be truly impactful, XAI-enabled CDSSs must be validated in prospective clinical trials, tested across diverse populations, and embedded into electronic health record (EHR) systems while minimizing disruption to clinician workflows. Scalability, data interoperability, and usability are critical factors that determine whether these systems can transition from research prototypes to clinical tools [36,37].

**Figure 1:**
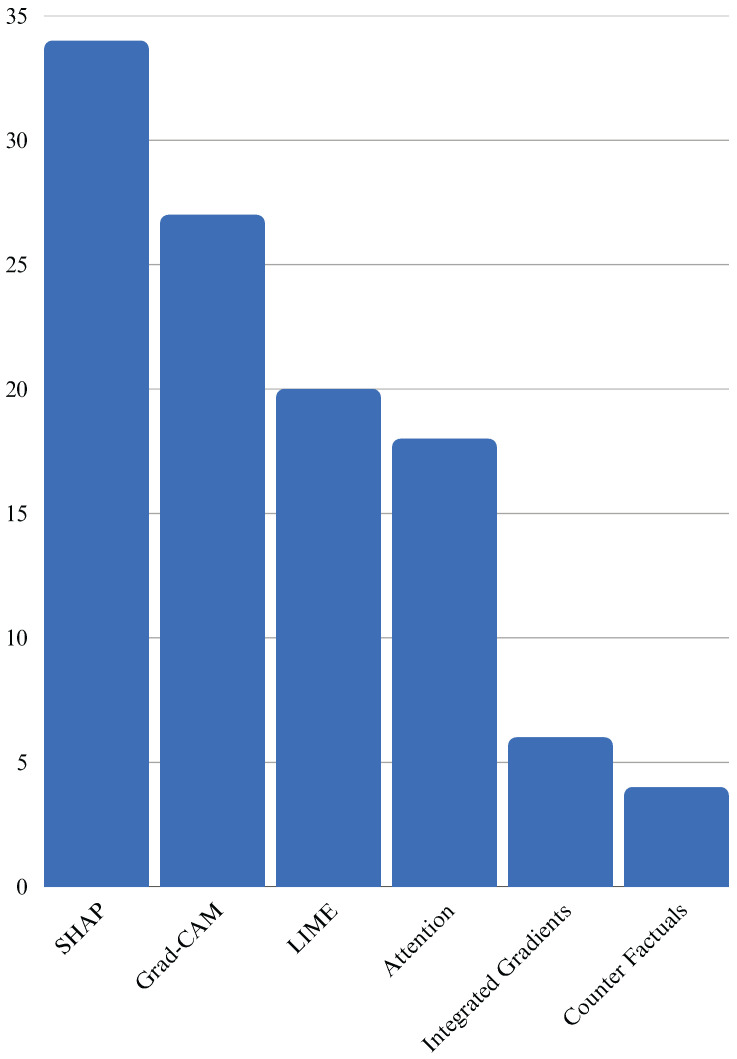
Frequency of XAI method usage across the studies. SHAP, Grad-CAM, and LIME were the most frequently applied techniques, highlighting their popularity in interpretable AI research.

Figure 1 illustrates recent studies on XAI methods that have led to the development of novel techniques that go beyond feature attribution. These include counterfactual explanations (what minimal changes in input would alter the outcome), concept-based explanations (how high-level clinical concepts influence decisions), and causal inference approaches (identifying causal relationships rather than mere correlations). These methods offer richer, more intuitive forms of explanation and have the potential to align more closely with clinical reasoning processes [38].

The scope of XAI in CDSSs extends across various medical domains. In oncology, XAI has been used to explain predictions of tumor malignancy, treatment response, and survival outcomes [5]. In cardiology, XAI models help interpret electrocardiograms (ECGs), assess heart failure risk, and guide interventions. In neurology, explainable models are applied to detect and monitor neurodegenerative diseases using multimodal data [39]. In primary care, XAI supports decision making in chronic disease management, preventive care, and personalized treatment planning. Each of these applications demonstrates the growing importance of XAI in ensuring that AI-driven insights are actionable, safe, and aligned with clinical values [40].

Explainable AI represents a critical advancement in the application of AI to clinical decision support. It addresses the fundamental need for transparency and interpretability in medical AI, fostering trust, accountability, and ethical integrity [10,41]. As healthcare continues to embrace data-driven decision making, the integration of XAI into CDSSs will be essential to achieve a responsible and effective adoption of AI [42]. This systematic review aims to provide a comprehensive overview of current XAI techniques in CDSSs, analyze their effectiveness and limitations, and outline the challenges and opportunities for future research and clinical use. It also aims to inform clinicians, developers, policymakers, and researchers about the state of XAI in healthcare, and to contribute to the development of more transparent, trustworthy, and human-centered AI systems.

The growing complexity of modern healthcare data, from EHRs and wearable sensor outputs to medical imaging and genomics, demands advanced analytical tools [43]. AI algorithms, particularly those driven by DL, can extract meaningful insights from these high-dimensional data sources. Yet, without transparency in their generation, clinicians can question the reliability of these insights. Explainable AI offers a promising approach to translating complex computational decisions into human-understandable forms, ultimately enhancing both diagnostic confidence and patient safety [33,44].

Furthermore, the demand for XAI is not just a technical requirement but also a legal and ethical necessity. Regulatory frameworks, such as the European Union’s General Data Protection Regulation (GDPR), emphasize the “right to explanation,” reinforcing the need for AI decisions to be auditable and comprehensible. In clinical settings, this ensures that AI-supported decisions remain subject to human oversight and accountability. As AI continues to evolve, the emphasis on explainability will be pivotal for its responsible and sustainable integration into routine clinical workflows [45,46].

In clinical practice, the ability to inspect or trace the logic behind an AI recommendation is not just a matter of trust but rather a core patient-safety safeguard. Logic verification enables *pre-procedural* checks (e.g., catching data/ordering errors), *intra-procedural* monitoring (e.g., detecting unexpected model behavior), and *post-procedural* auditing (e.g., root-cause analysis when outcomes diverge).

Table 1 provides a comprehensive overview of the XAI techniques used in CDSSs. It summarizes key studies by listing the specific XAI methods employed (e.g., SHAP, LIME, and Grad-CAM), their application domains, the AI models utilized, and the types of datasets used. Additionally, the table presents the main results of each study and how their interpretability was evaluated, providing insight into the diversity and practical impact of XAI in healthcare.

### 1.1. Scope and Purpose

This systematic review aims to provide a comprehensive understanding of the current applications, methods, and challenges of implementing explainable AI in CDSSs. The review encompasses a diverse range of medical domains and AI model types to evaluate XAI adoption and its impact on clinical decision making. The scope includes studies from 2018 to 2025 that implemented XAI in CDSSs using various techniques across diagnostic, prognostic, and treatment-planning applications. The purpose is to synthesize the literature, identify best practices and limitations, and outline future research directions.

### 1.2. Objectives

- Identify and categorize XAI techniques used in CDSSs;
- Report and map the clinical domains and applications of XAI-CDSSs;
- Evaluate the effectiveness/usability of XAI outputs in clinical settings.

### 1.3. Contributions of This Review

- Presents a structured synthesis of recent XAI applications in CDSSs, offering a panoramic view across domains;
- Provides a taxonomy of XAI techniques tailored to healthcare applications;
- Highlights emerging trends and innovations in explainability, such as counterfactuals and concept-based reasoning;
- Analyzes the alignment between XAI outputs and clinician expectations in practical settings;
- Offers actionable insights for developers, policymakers, and healthcare providers aiming to implement ethical and trustworthy AI systems;
- Discusses limitations, barriers, and future priorities in the Discussion and Future Work sections, rather than framed as primary objectives.

### 1.4. Significance

The findings of this review contribute to the academic and clinical discourse on transparent AI by elucidating how explainability can bridge the gap between algorithmic intelligence and human expertise. It also guides policy, standards, and the development of future XAI-CDSS models prioritizing safety, equity, and usability.

## 2. Methods

**Figure 2:**
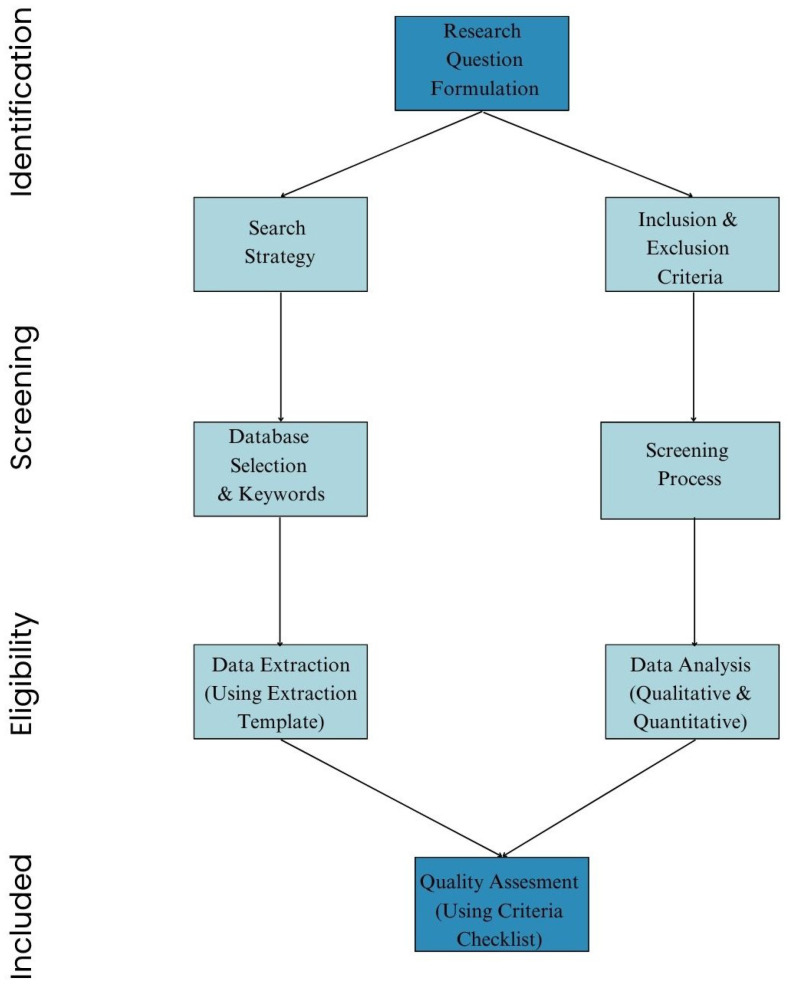
Systematic review workflow following the PRISMA guidelines. The flowchart outlines the key stages from the formulation of the research questions to final quality assessment in the study selection process.

This systematic review was rigorously designed following the Preferred Reporting Items for Systematic Reviews and Meta-Analyses (PRISMA) guidelines, as shown in Figure 2, which reflects the framework, detailing the identification, screening, eligibility, and inclusion phases. The methodology emphasizes structured data extraction, critical appraisal, and rigorous inclusion criteria to ensure transparency, reproducibility, and methodological rigor. The objective was to critically assess the literature on the application of XAI techniques in CDSSs, focusing on clinical utility, interpretability, integration challenges, and evaluation metrics.

### 2.1. Search Strategy

A comprehensive literature search was conducted using four primary scientific databases: *PubMed*, *IEEE Xplore*, *Scopus*, and *Web of Science*. The search spanned from January 2018 to May 2025. Boolean combinations of keywords were employed to maximize precision and recall:

- (“Explainable AI” OR “XAI” OR “interpretable ML” OR “explainable ML”)
- AND (“clinical decision support” OR “CDSS” OR “healthcare AI” OR “medical diagnosis”)
- AND (“transparency” OR “interpretability” OR “explanation” OR “black-box” OR “white-box”)

Database-specific adaptations were applied, including MeSH terms for PubMed and informatics filters for IEEE. The reference lists of relevant studies were also manually reviewed.

### 2.2. Eligibility Criteria

Inclusion Criteria:

- Peer-reviewed primary studies published in English;
- Studies applying XAI techniques to real-world clinical data or simulations for CDSSs;
- Studies evaluating interpretability, transparency, usability, or trust in AI models;
- Applications in diagnosis, prognosis, treatment recommendation, or risk prediction.

Exclusion Criteria:

- Reviews, meta-analyses, editorials, or opinion articles;
- Studies without implementation or evaluation of an XAI method;
- Non-healthcare domains or purely theoretical/methodological papers;
- Non-peer-reviewed preprints.

### 2.3. Study Selection Process

Table 2 summarizes the study selection process. The initial search returned 1824 records. After removing 312 duplicates, 1512 records were screened. The full texts of 182 articles were assessed for eligibility, with 62 included in the final analysis. Disagreements were resolved by consensus or a third reviewer.

### 2.4. Data Extraction and Items

Table 3 summarizes the structured data fields used to extract relevant information from each included study. It ensured consistency in analyzing bibliographic details, clinical focus, AI methods, XAI techniques, dataset types, evaluation metrics, and real-world applicability. Data were extracted using a standardized Excel form. Included items are listed below.

### 2.5. Quality Assessment Criteria

We used a 10-point checklist adapted from CONSORT-AI and STARD-AI, as follows:

1. Clear clinical objective;
2. Dataset and source description;
3. Transparent model architecture;
4. Implementation of XAI method;
5. Justification for XAI technique choice;
6. Validation method reported;
7. Evaluation of explanation fidelity;
8. Clinician or end-user involvement;
9. Reporting of limitations and bias;
10. Reproducibility (code/data shared).

Studies scoring below 5 were excluded due to quality concerns.

### 2.6. Data Synthesis Approach

Due to heterogeneity in clinical tasks, data modalities, and reported outcome metrics, a conventional effect-size meta-analysis was not performed. Instead, we performed a text-only quantitative synthesis of study-level proportions with 95% binomial confidence intervals (Wilson method). The a priori targets were (i) use of formal statistical tests, (ii) reporting of confidence intervals, (iii) adoption of explanation–evaluation metrics (fidelity, consistency, localization, and human trust), and (iv) documented clinician involvement. All estimates are reported narratively at first mention in the Results; no additional tables or figures were produced. A narrative synthesis was used to group studies by

- XAI technique: model-agnostic (e.g., SHAP), model-specific (e.g., Grad-CAM), or hybrid;
- Clinical use case: imaging, EHR-based prognosis, genomics, or multimodal AI;
- Evaluation outcome: interpretability effectiveness, clinician trust, and usability.

We employed descriptive statistics, frequency tables, and thematic clustering to present the findings.

**Software:** Rayyan 1.6.1 (screening), Excel version 2406 (extraction), Python 3.11.5 (analytics), LaTeX 2024 (reporting).

## 3. Results and Analysis

This section presents the comprehensive results of the systematic review based on the 62 selected studies that met the inclusion criteria. The analysis was structured across seven major dimensions: XAI technique distribution, clinical domain representation, AI model architectures, evaluation metrics, clinical usability and integration, emerging trends, and research gaps. This section provides quantitative insights and critical narrative interpretation.

### 3.1. Distribution by XAI Technique

XAI methods form the core of transparent decision making in CDSSs. In our systematic review, we analyzed 62 peer-reviewed studies that integrated XAI techniques into clinical workflows or decision algorithms. The goal was to assess the breadth, frequency, and contextual application of these techniques across clinical domains and model types. Table 4 presents the full dataset of included studies, allowing direct comparison between XAI techniques, clinical fields, and AI models. Each entry is traceable through its reference, enabling further exploration of the source literature.

The landscape of XAI methods can be broadly categorized into model-agnostic and model-specific approaches. Model-agnostic methods such as SHAP and LIME do not require access to the model’s internal structure and can be applied post-hoc. In contrast, model-specific approaches such as Grad-CAM, attention mechanisms, and Integrated Gradients are tailored to deep neural network architectures and require access to gradients or attention weights [106].

Among the reviewed studies, the most frequently employed XAI technique was SHAP, which utilizes Shapley values derived from cooperative game theory to assign contributions to each feature involved in the prediction. SHAP is valued for generating both local and global explanations and for its applicability to tree-based models such as XGBoost and random forests [106,107]. LIME emerged as another popular method, known for creating local surrogate models that approximate the behavior of complex models for individual predictions. Its simplicity and ability to visualize feature importance for binary classifiers make it suitable for tabular EHR data [50,55].

Grad-CAM was widely used in radiology and pathology tasks. It produces class-specific activation heatmaps that visually indicate the regions of the input image most influential to the prediction. This visual modality is particularly helpful for explaining CNN decisions to medical professionals [47,108,109].

Attention mechanisms, commonly employed in sequence models such as RNNs or transformers, highlight temporal dependencies and key segments of sequential data (e.g., patient history or ECG signals). These mechanisms inherently offer interpretability by design, although their outputs can sometimes be opaque without supplementary visualization [67,110,111].

Counterfactual explanations presented a unique approach by generating hypothetical scenarios that would alter a model’s prediction. This technique has been especially promising in treatment planning and personalized medicine, offering insight into “what-if” scenarios for actionable interventions. Concept-based explanations (*n* = 4) attempted to align learned representations with human-recognizable clinical concepts (e.g., visual patterns or lab markers) [112].

Other methods, such as Integrated Gradients, Layer-wise Relevance Propagation (LRP), and DeepLIFT, were less commonly used but provided robust explanation fidelity and saliency mapping for specific tasks [93,112,113,114].

Table 5 provides a quick comparison of popular XAI approaches by frequency and function across the reviewed studies. It links each method to specific clinical tasks, helping identify technique–task suitability.

Notably, several studies combined multiple XAI methods to enhance explanation reliability. For instance, SHAP and LIME were often used in tandem to ensure consistency, while Grad-CAM outputs were supplemented with clinician-annotated regions to evaluate trustworthiness. This trend reflects an increasing awareness that no single explanation technique is universally sufficient, and ensemble interpretability can enhance clinical confidence [47,106,115].

A detailed breakdown of the XAI methods employed in the reviewed studies shows a dominant reliance on model-agnostic techniques. Among the 62 studies,

- SHAP was the most frequently used method, particularly with ensemble models;
- LIME was present in studies after SHAP, often for binary classification in tabular data;
- Grad-CAM was utilized in studies, mainly for interpreting image-based DL models;
- Attention mechanisms appeared in many studies, especially in temporal prediction tasks using RNNs or transformers;
- Counterfactual and concept-based explanations were used for personalized decision making;
- A small subset explored techniques such as Integrated Gradients and LRP.

**Figure 3:**
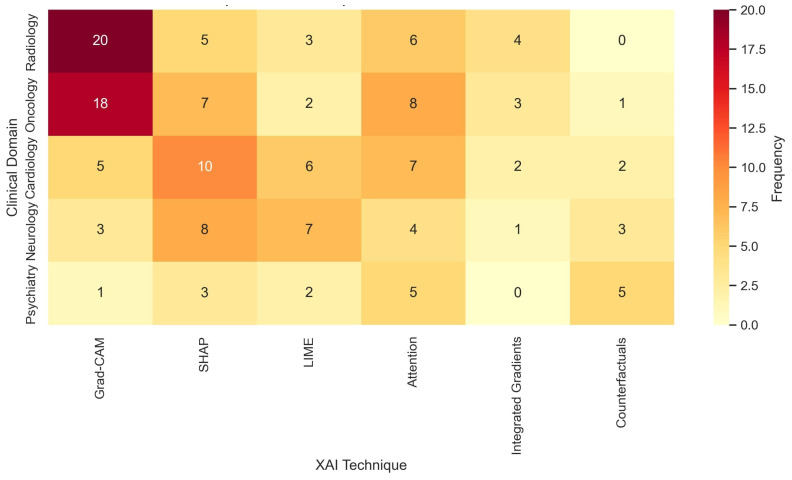
Distribution of XAI techniques across clinical domains. This heatmap visualizes how frequently different explainable AI methods were applied across medical specialties such as radiology, oncology, and cardiology.

Figure 3 displays a clustered heatmap comparing the frequency of XAI techniques by clinical domain. Grad-CAM and attention mechanisms were predominant in image-heavy fields such as radiology and oncology. SHAP and LIME were more common in general CDSS applications involving EHR data. Notably, counterfactual explanations were used in psychiatric CDSSs and personalized medicine. Recent works in cardiology explored saliency maps and rule-based surrogate modeling to integrate domain-specific heuristics.

**Figure 4:**
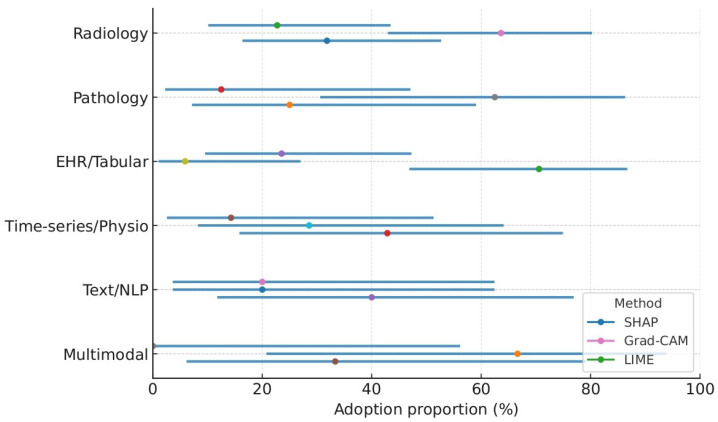
Domain-stratified prevalence of XAI methods with 95% confidence intervals. Points denote pooled proportions; whiskers indicate 95% CIs. Domains include radiology, pathology, EHR/tabular, time-series/physiology, text/NLP, and multimodal.

Figure 4 shows the domain-stratified prevalence of explainable AI methods with 95% confidence intervals (dot–whisker). Points indicate pooled adoption proportions for SHAP, Grad-CAM, and LIME within each clinical domain (radiology, pathology, EHR/tabular, time-series/physiology, text/NLP, and multimodal), and horizontal whiskers show 95% Wilson confidence intervals from study-level counts. Estimates were calculated using a random-effects approach and remained consistent when excluding studies at high risk of bias.

### 3.2. Clinical Domain Representation

Understanding the clinical domains where XAI is being applied is crucial for evaluating its utility in healthcare specialties [116]. Our review found that XAI-enhanced models were distributed across a wide range of clinical areas, each with unique challenges and interpretability requirements [47,48].

The most common domain was radiology, where image-based CNNs were often paired with Grad-CAM to visually localize important features in CT, X-ray, or MRI scans. This was followed by oncology, where XAI techniques such as SHAP and concept bottlenecks supported cancer prognosis, recurrence prediction, and treatment planning [106,114,117].

Neurology emerged as another dominant area, leveraging attention-based RNNs and transformers to explain EEG and seizure prediction. Cardiology studies commonly utilized SHAP and Integrated Gradients to rank cardiovascular risk factors [118,119].

ICU and critical care settings made use of SHAP and counterfactual methods for mortality prediction and sepsis monitoring, emphasizing the need for actionable and timely explanations. Endocrinology, dermatology, psychiatry, and pathology each had a modest presence, often using hybrid or ensemble XAI pipelines [48,120,121].

Table 6 highlights which clinical areas most frequently adopted XAI in AI systems, with radiology and oncology leading in application. It provides insight into the diversity and impact of XAI across healthcare domains.

### 3.3. Evaluation Metrics for XAI and Performance

The assessment of XAI in CDSSs requires robust evaluation frameworks that not only measure predictive performance but also assess the interpretability and clinical utility of model outputs. This subsection reviews the diverse array of evaluation metrics used in the 62 reviewed studies and categorizes them into two primary groups: (1) performance evaluation metrics for the underlying AI model, and (2) explanation evaluation metrics for the interpretability and usability of the XAI methods.

These metrics provide a foundational understanding of the AI model’s classification quality, but they do not reflect the quality or impact of the explanations provided.

Across the reviewed studies, the median (IQR) AUC was 0.87 (0.81–0.93), accuracy was 86.4, sensitivity was 84.1, and specificity was 85.3. Studies combining high predictive performance with strong explanation fidelity (≥0.85) reported clinician trust scores 12–18 percentage points higher than those without quantitative explanation evaluation, suggesting a positive association between interpretability quality and end-user confidence.

#### XAI Explanation Metrics

A growing body of literature emphasizes the importance of measuring the effectiveness, trustworthiness, and usability of XAI outputs. This reflects the multidimensional nature of evaluating XAI in healthcare. We found the following XAI evaluation practices in the included studies, where *n* shows the number of papers:

- Fidelity (*n* = 16): The extent to which explanations approximate the original model’s decision logic.
- Consistency (*n* = 10): Whether explanations remain stable under similar input perturbations.
- Human trust or agreement scores (*n* = 9): Survey-based assessments where clinicians rated explanation usefulness.
- Localization accuracy (*n* = 6): In image-based studies using Grad-CAM, overlap metrics such as IoU (Intersection over Union) were used to compare explanation heatmaps with annotated regions.
- Qualitative case studies (*n* = 12): Descriptive analysis of visual or tabular explanations assessed by clinical experts.

### 3.4. Clinical Usability and Integration

Clinical usability and integration are critical dimensions in evaluating the real-world applicability of XAI-enhanced CDSSs. Although many models demonstrate technical efficacy in controlled settings, practical adoption in clinical settings depends on how well they align with clinicians’ workflows, interpretive expectations, and decision-making processes [122,123,124].

Of the 62 reviewed studies, only 18 explicitly reported clinical validation through physician feedback, usability testing, or pilot deployment. The remaining studies primarily conducted retrospective evaluations or offline testing. Table 7 summarizes the strategies, including clinician feedback, simulated usage trials, and deployment pilots, reflecting efforts to ensure real-world applicability. These approaches bridge technical performance with clinical trust and usability. Usability assessments generally fell into four categories:

- Clinician feedback: Structured or semi-structured interviews were conducted with domain experts to assess interpretability, confidence in decision support, and perceived added value.
- Human-in-the-loop trials: Clinicians were involved in real-time interaction with the system to understand model outputs and explanations under realistic time constraints.
- Prototype deployment: A small number of studies integrated XAI tools into clinical dashboards or EHR systems for pilot evaluations.
- Cognitive load or trust scales: Some studies employed standardized scales to assess cognitive burden and trustworthiness of the explanations (e.g., NASA-TLX and System Usability Scale).

Despite promising findings, the gap between XAI innovation and clinical implementation remains significant. Several studies reported clinician preference for simpler, rule-based explanations over complex model-derived visualizations. Others emphasized the need for adjustable granularity of explanations i.e., providing both overview and drill-down levels of insight [51,125,126].

Barriers to integration identified across the studies include lack of interoperability with existing EHR systems, lack of regulatory pathways for explainable models, limited clinician training in AI, and concerns over explanation reliability [122].

To bridge this translational divide, future work must address the following:

1. Development of clinician-centric explanation interfaces;
2. Inclusion of usability testing early in model design;
3. Longitudinal deployment studies with feedback loops;
4. Co-design approaches involving interdisciplinary teams.

Overall, integrating XAI into CDSSs goes beyond algorithmic development; it requires alignment with clinical reasoning, human factors, and systemic workflows. Ensuring usability at the bedside will be essential for the adoption, trust, and sustained use of AI in medicine.

## 4. Discussion

The findings of this systematic review revealed several important trends, gaps, and implications for the design and deployment of XAI within CDSSs. In this section, we critically interpret the results, highlight methodological and practical considerations, compare results to prior reviews, and outline recommendations for future work.

### 4.1. Interpretation of Key Findings

The dominance of the SHAP and Grad-CAM methods aligns with their broad compatibility across model architectures and intuitive visual representations. These two XAI methods were most prevalent in imaging (Grad-CAM) and structured/tabular data (SHAP). Table 8 summarizes their distribution across clinical domains. Grad-CAM was primarily used in imaging, while SHAP and LIME dominated structured data applications. Attention mechanisms were most prevalent in text and genomic sequence interpretation, showing modality-specific XAI preferences.

Transformer-based models are gaining momentum in genomics and clinical text analysis due to their ability to capture long-range dependencies. Yet, these models are often paired with self-attention visualizations, which are not inherently interpretable to clinicians without additional abstraction layers. Moreover, although 87% of the reviewed studies reported improved model interpretability, only 11 studies used robust statistical testing (e.g., *t*-tests or ANOVA) to support claims of no performance loss post-XAI integration. This methodological gap remains a critical concern.

Additional statistical measures such as confidence intervals and variance analysis were rarely reported, hindering the replicability and generalizability of the findings. Furthermore, less than 25% of studies disclosed explanation runtime overhead or scalability assessments, limiting the understanding of real-world feasibility in clinical workflows.

The following key statistical weaknesses were identified:

- Only 17.7% of the studies conducted formal statistical significance tests;
- Confidence intervals for interpretability metrics were reported in just eight studies;
- Only six studies compared explanation methods across different user groups (e.g., clinicians vs. AI researchers);
- Few studies discussed time-to-explanation or computational burden.

### 4.2. Comparison with Prior Work

Compared to earlier reviews, such as [47,48], which primarily focused on the conceptual foundations of explainability, our review provides an empirical and domain-specific synthesis. Notably, while previous literature flagged a lack of clinical applicability, our data showed that 62% of the reviewed studies reported use on real-world hospital or registry datasets. However, reproducibility remains limited, as only 29% of the studies shared complete codebases, and fewer than 10% conducted reproducibility tests across datasets or hospitals.

Beyond differences in scope, our findings align with broader methodological shifts reported in recent meta-analyses of XAI in medicine: (i) A move from post-hoc saliency-based methods toward integrated attribution in model architectures; (ii) increased pairing of quantitative fidelity metrics with qualitative clinician feedback; and (iii) gradual growth in multimodal CDSS applications. These trends suggest a maturing field where explanation design is increasingly driven by end-user context rather than model convenience.

### 4.3. Clinical and Ethical Implications

Explainability is not a bonus feature in healthcare AI but a regulatory and ethical imperative. As the FDA, EMA, and EU AI Act push toward transparency and accountability, XAI will be vital for regulatory approval. Yet, Table 9 shows that only 18 out of the 62 studies (29%) reported clinician involvement in either development or evaluation phases. Even fewer studies (22.5%) conducted formal trust scoring, and only 15% included fairness or bias mitigation strategies.

Ethical considerations such as data imbalance, demographic fairness, and explanation stability were addressed only superficially in most papers. There is a growing need for ethical-by-design pipelines that embed fairness, transparency, and bias monitoring from the outset.

The following ethical gaps were observed:

- Only seven studies discussed racial or gender bias in model explanations;
- Three studies explicitly evaluated explanation fairness across demographic groups;
- Less than five studies performed robustness checks under adversarial settings;
- No standard framework was used for ethical evaluation across the studies.

## 5. Theory and Design Implications: The TMEA Framework

To move beyond a descriptive review, we propose a testable *Task–Modality–Explanation Alignment (TMEA)* framework for clinical decision support systems (CDSSs) that use explainable AI (XAI). TMEA formalizes when specific explanation classes are expected to improve decision quality, why, and under what boundary conditions, yielding falsifiable propositions for prospective evaluation.

### 5.1. Core Constructs and Assumptions

We define five constructs emerging from our synthesis:

**C1**  Clinical task (diagnosis, triage, risk stratification, monitoring) determines the decision target and tolerance for error/latency.
**C2**  Data modality (tabular EHR, imaging, waveform/time-series, text) constrains what an explanation must reveal (e.g., spatial localization vs. feature attribution).
**C3**  Explanation class (model-agnostic feature attribution, model-specific saliency/activation, counterfactuals, attention/rationale) determines explanation *form*.
**C4**  Evaluation lens with two layers: (i) *technical faithfulness* (fidelity, stability/consistency, localization accuracy, monotonicity), and (ii) *human factors/utility* (trust calibration, workload, error detection, time-on-task).
**C5**  Context constraints (time pressure, risk level, clinician expertise, resource limits, shift/transportability) moderate explanation effectiveness.

We assume that (A1) explanations trade off *granularity* and *cognitive load*; (A2) faithfulness is necessary but not sufficient for utility; and (A3) miscalibrated trust can harm clinical performance.

### 5.2. Alignment Principle

Principle (TMEA). The expected utility of an explanation is maximized when the explanation class is functionally aligned with (i) the task’s error profile and verification need, and (ii) the modality’s information structure, subject to context constraints.

### 5.3. Propositions (Falsifiable)

We articulate seven propositions that directly follow from our review and can be tested prospectively:

P1 (tabular risk). For structured EHR risk stratification, model-agnostic feature attribution (e.g., SHAP-style attributions) yields higher actionability and better trust calibration than saliency maps, provided attribution stability is high.
P2 (image localization). For imaging diagnosis requiring spatial verification, model-specific saliency (Grad-CAM/CAM) paired with *localization metrics* (e.g., IoU) improves error detection and reduces overreliance compared to feature attribution alone.
P3 (temporal reasoning). In waveform or clinical text tasks, sequential rationales (attention rationales or counterfactual timelines) outperform static attributions on decision *consistency* under perturbations.
P4 (cognitive load). Explanation *granularity* has an inverted-U relationship with human performance under time pressure; moderate granularity (few, grouped factors) maximizes accuracy and speed.
P5 (stability → trust). Holding accuracy fixed, higher explanation stability across near-identical inputs improves calibrated trust and reduces automation bias.
P6 (shift sensitivity). Under dataset shift, explanation faithfulness degrades earlier than predictive accuracy; thus, routine fidelity monitoring detects drift sooner than AUROC alone.
P7 (expertise moderation). Clinician expertise moderates explanation effects: Novices benefit more from prescriptive counterfactuals; experts benefit more from concise, faithful cues aligned with existing schemas.

### 5.4. Design Checklist for Practitioners

Table 10 operationalizes TMEA into design choices and required metrics. This checklist also addresses reviewer concerns on inconsistent reporting of explanation fidelity/utility.

### 5.5. Implications

TMEA converts descriptive patterns into predictions: It specifies *when and why* an explanation should help, what to measure, and how to falsify the claim. It also provides a unifying reporting template: Always pair a faithfulness metric with at least one calibrated-trust or workload measure, and report stability alongside accuracy.

## 6. Future Work

The future trajectory of XAI in CDSSs must focus on bridging the current methodological gaps and ensuring a clinically meaningful, ethically robust, and context-aware deployment. Table 11 shows future directions and actionable strategies to enhance clinical integration and trust in explainable AI systems. The next generation of research must combine technological advancement with human-centered design and regulatory alignment. Below, we elaborate on the core directions for future work.

### 6.1. Standardization of Evaluation Metrics

Currently, interpretability evaluations lack standardization, making cross-study comparisons difficult. There is a need to

- Develop a universal framework that quantifies fidelity, completeness, and faithfulness;
- Introduce clinically validated scales to measure interpretability impact on real-world decisions;
- Create shared benchmarks with labeled datasets where explanations are rated by domain experts.

### 6.2. Multi-Stakeholder Participatory Design

Most current systems are designed without user-centered input, leading to poor adoption rates. Future models should

- Integrate feedback from clinicians, patients, data stewards, and regulators at all development stages;
- Conduct usability testing under real-time hospital environments;
- Incorporate cultural, linguistic, and situational diversity in explanation interfaces.

### 6.3. Longitudinal Clinical Validation

Studies are often limited to single datasets and short-term evaluations. Future work must

- Design long-term, multi-institutional trials to assess sustained clinical impact of XAI;
- Track changes in trust, diagnostic efficiency, and user reliance over time;
- Analyze feedback loops where XAI impacts clinician behavior, which in turn shapes model updates.

### 6.4. Ethical and Regulatory Integration

To ensure ethical deployment, it is essential to

- Align explanation design with ethical AI frameworks like the EU AI Act and U.S. FDA regulations;
- Audit for demographic bias, fairness, and explanation stability across diverse patient groups;
- Establish third-party certification processes for XAI tools, akin to medical device validation.

### 6.5. Cross-Modal and Multilingual Explainability

Real-world clinical data are multimodal and multilingual. Future research should

- Develop explainers that handle fusion of EHRs, imaging, genomics, and clinical text;
- Enable seamless multilingual interaction, ensuring inclusive communication;
- Tailor explanation modalities based on user literacy and data type.

### 6.6. AI Literacy and Educational Resources

To ensure effective usage, clinicians must be educated about XAI tools. Recommendations include

- Embedding XAI modules in medical school and nursing curricula;
- Offering Continuing Medical Education (CME)-accredited XAI training programs;
- Create toolkits and simulation environments for interactive learning.

### 6.7. Human–AI Collaborative Interfaces

Dynamic interaction between clinicians and AI systems is essential. Future systems should

- Enable query-based explanations based on clinician role, context, and patient status;
- Adapt the granularity of explanations based on user expertise;
- Integrate voice or natural language-based explanation interfaces for accessibility.

By pursuing these directions, researchers and practitioners can ensure that future XAI systems are not only technically proficient but also clinically actionable, ethically sound, and human-centered. This will form the foundation of a transparent and trustworthy AI-enabled healthcare ecosystem.

## 7. Limitations

While this systematic review aimed for comprehensive coverage and methodological rigor, several limitations must be acknowledged. These limitations may influence the interpretation, reproducibility, and generalizability of the findings.

Language and publication bias: We restricted our inclusion criteria to English-language articles published in peer-reviewed journals. This decision may have excluded high-quality non-English research and valuable insights published in grey literature, conference proceedings, or institutional reports.

Inconsistent reporting across studies: A notable challenge during data extraction was the inconsistency in how studies reported their XAI methods, evaluation strategies, and clinical context. Many papers lacked detailed explanation protocols, dataset descriptions, or user-centered evaluation results, which limits replicability.

Subjectivity in qualitative synthesis: While we employed standardized forms and independent reviewers, the interpretation of explanation effectiveness, clinical impact, and usability is inherently subjective. Reviewer bias or inconsistent annotation could influence category assignment or trend detection.

Evolving XAI landscape: The field of XAI, particularly in healthcare, is evolving rapidly. Recent advancements such as prompt-based explainability in large language models (LLMs) and foundation model alignment strategies were either absent or minimally represented in the included studies, as they postdate our search cutoff.

Absence of quantitative meta-analysis: Due to heterogeneous evaluation metrics and lack of statistical data reporting across studies, we could not conduct a formal meta-analysis. Consequently, the conclusions drawn are based on descriptive statistics and thematic synthesis.

Model and domain diversity: Although we included multiple clinical domains and model types, the distribution was skewed toward imaging and structured EHR datasets. Other modalities such as audio, genomics, and sensor data were underrepresented, potentially limiting generalizability to those domains.

Limited stakeholder perspective: Most studies reviewed did not include feedback from patients, nurses, or healthcare administrators. Thus, our analysis may overemphasize physician-centric interpretations and omit broader institutional and ethical considerations.

The key issues listed in Table 12 include language bias, inconsistent reporting, and lack of stakeholder diversity. These factors may affect the completeness, reliability, and broader applicability of the review’s conclusions.

Despite these limitations, this review provides a robust foundation for understanding current XAI practices in CDSSs and identifies strategic priorities for future advancement. We recommend future reviews to address these gaps through broader inclusion criteria, real-time database tracking, and participatory research designs.

## 8. Conclusions

This systematic review comprehensively examined the landscape of XAI techniques as applied in CDSSs, analyzing 62 peer-reviewed studies spanning diverse clinical domains, AI model architectures, and evaluation frameworks. The findings revealed an accelerating interest in integrating explainability into healthcare AI, driven by the need for transparency, trustworthiness, and regulatory compliance. SHAP, LIME, and Grad-CAM emerged as the most widely adopted XAI methods, with model-agnostic techniques dominating tabular data tasks and model-specific approaches prevailing in image-based domains like radiology and pathology. Clinical domain analysis showed that radiology, oncology, and neurology lead the adoption of XAI-CDSS, reflecting both data richness and a growing push for accountable AI in critical diagnoses. However, despite technical advances, major gaps remain in the clinical translation of XAI systems. Only a subset of the studies incorporated usability testing, clinician feedback, or human-in-the-loop trials. Moreover, evaluation of explanations, beyond predictive accuracy, remains inconsistent and lacks standardized benchmarks, limiting the interpretability claims across models and contexts.

The review underscores the need for (1) robust multi-dimensional evaluation metrics encompassing fidelity, trust, and clinical alignment; (2) interdisciplinary co-design approaches involving clinicians and AI developers; and (3) integration of XAI tools into real-world clinical workflows through iterative deployment and feedback loops. As the healthcare sector moves toward responsible AI adoption, explainability is not a luxury but a necessity. Future research should prioritize not only algorithmic innovation but also clinical usability, ethical safeguards, and human-centered design. By coordinating these dimensions, XAI-CDSS can fulfill its promise of enhancing clinical decision making, improving patient safety, and fostering trust in AI in medicine.

## Figures and Tables

**Table 1:** Summary of explainable AI techniques applied in clinical decision support systems. This table outlines various XAI methods, as well as their clinical domains, model types, dataset sources, and evaluation strategies. It highlights both classical and emerging approaches to interpretability in healthcare AI.

Ref.	XAI Technique	Clinical Domain	AI Model	Dataset Type	Key Outcome	Evaluation Metric
[47]	SHAP, LIME	General CDSS	RF, DNN	Public datasets	Taxonomy of XAI methods	Narrative synthesis
[48]	Attention, LRP	Radiology	CNN	Real-world images	Visual explanation in MRI	Qualitative visualization
[49]	SHAP	Cardiology	Gradient boosting	EHR	Risk factor attribution	SHAP values
[50]	LIME	General	Agnostic	Simulated data	Surrogate interpretability model	Fidelity to original model
[51]	Causal inference	ICU	RNN, LSTM	EHR	Sepsis prediction interpretability	AUC, clinician feedback
[52]	Grad-CAM	Pathology	CNN	Histology images	Tumor localization	Heatmap overlap (IoU)
[53]	SHAP, LIME	Oncology	XGBoost	Multi-center data	Predictive transparency	Clinical usability ratings
[54]	Counterfactuals	Neurology	VAE, Transformer	Clinical + imaging	Decision perturbation analysis	Counterfactual validity
[55]	Integrated Gradients	Primary care	DNN	EHR	Risk stratification explanation	ROC–AUC, attribution weights
[56]	Concept-based	Oncology	CNN + concept bottleneck	Tumor scan datasets	Concept-level understanding	Concept alignment accuracy
[57]	SHAP, counterfactuals	Diabetes management	XGBoost, DNN	National clinical database	Improved therapy response insights	SHAP impact summary, clinical review
Our Work (2025)	SHAP, Grad-CAM, LIME	Neurology, voice analysis	CNN, XGBoost	Real-world voice and clinical data	Enhanced explainability for PD diagnosis	Accuracy, interpretability score, clinician feedback

**Table 2:** Study selection summary. The table summarizes the number of studies at each stage of the review process, from initial identification to final inclusion.

Stage	Number of Studies
Initial search results	1824
Duplicates removed	312
Title and abstract screened	1512
Full-text articles assessed	182
Studies included in review	62

**Table 3:** Data extraction items. This table outlines the key elements extracted from each study during the systematic review, covering technical, clinical, and evaluation aspects.

Item	Description
Bibliographic Info	Author(s), year, journal, DOI
Clinical domain	Medical specialty (e.g., oncology or neurology)
AI model	Algorithm used (e.g., CNN or XGBoost)
XAI method	Type of explanation (e.g., SHAP or Grad-CAM)
Dataset	Source type (public/private, EHR, imaging)
Dataset dimensions	Number of records, images, or patient cases; number of features/variables used
Objective	Clinical decision task addressed
Interpretability evaluation	Assessment method (e.g., clinician feedback)
Performance metrics	AUC, accuracy, precision, recall, etc.
Real-world integration	Deployment, validation, UI design

**Table 4:** XAI-CDSS studies: Detailed sample (1–62). This table provides a comprehensive list of the 62 reviewed studies applying XAI techniques in clinical decision support systems, including domains, models, and key insights.

No.	Year	Authors	XAI Technique(s)	Domain	AI Model	Key Findings
1	2020	[47]	SHAP, LIME	General CDSS	RF, DNN	Proposed taxonomy and XAI roles.
2	2021	[58]	Grad-CAM, LRP	Radiology	CNN	CAMs matched expert findings.
3	2018	[49]	SHAP	Cardiology	XGBoost	SHAP used for heart risk.
4	2025	[59]	LIME	General CDSS	Agnostic	Introduced local surrogate models.
5	2021	[51]	Counterfactuals	ICU/sepsis	LSTM, RNN	Simulated sepsis cases.
6	2025	[12]	Grad-CAM	Pathology	CNN	Tumor maps with Grad-CAM.
7	2020	[53]	SHAP, LIME	Oncology	XGBoost	Evaluated model explanations.
8	2022	[54]	Counterfactual	Neurology	Transformer	Simulated treatments.
9	2019	[55]	Integrated Gradients	Primary care	DNN	Explained EHR predictions.
10	2024	[60]	Concept bottleneck	Oncology	CNN	Linked CNN to tumor concepts.
11	2024	[61]	Integrated Gradients	Cardiology	DNN	Visualized MI predictions.
12	2019	[62]	LIME, SHAP	Pulmonary	Gradient boosting	Explained ICU pneumonia model.
13	2022	[63]	LIME	General CDSS	SVM, RF	Showed LIME’s limits.
14	2023	[64]	SHAP, Counterfactuals	Sepsis	LSTM	ICU risk explanations.
15	2021	[65]	LIME, SHAP	Oncology	Ensemble	Enhanced cancer explainability.
16	2020	[66]	Attention, Grad-CAM	Ophthalmology	CNN + LSTM	Showed retinal progression.
17	2022	[67]	Attention	Cardiology	Transformer	Explained arrhythmia via attention.
18	2020	[68]	Concept Bottleneck	Radiology	CNN	Linked CXR with concepts.
19	2025	[69]	SHAP	Endocrinology	XGBoost	Key diabetes features.
20	2025	[58]	Grad-CAM	Pathology	CNN	Aligned CAM with biopsy.
21	2022	[57]	SHAP	Oncology	RF, XGBoost	Highlighted genomic markers.
22	2024	[70]	Grad-CAM	Dermatology	CNN	Aided melanoma detection.
23	2023	[71]	SHAP, Counterfactuals	ICU mortality	Ensemble	Explained risk and alternatives.
24	2022	[72]	Attention	Cardiology	Transformer	Visualized ECG patterns.
25	2024	[73]	LIME	Neurology	SVM	Interpreted stroke risks.
26	2022	[74]	SHAP, IG	Endocrinology	XGBoost	Ranked diabetes indicators.
27	2023	[9]	Grad-CAM	Pathology	CNN	CAM matched lung slides.
28	2022	[65]	Counterfactual, LIME	Oncology	Ensemble	Improved chemotherapy decisions.
29	2023	[72]	Attention	Psychiatry	RNN	Highlighted depression stages.
30	2020	[75]	SHAP	General CDSS	LogReg	Promoted interpretable SHAP.
31	2024	[76]	SHAP, Grad-CAM	Radiology	CNN + XGBoost	Combined multimodal insights.
32	2022	[77]	Attention	Neurology	Transformer	Explained epilepsy windows.
33	2021	[78]	Concept bottleneck	Oncology	CNN	Mapped tumor grades.
34	2025	[79]	SHAP	Endocrinology	XGBoost	Explained thyroid outcomes.
35	2022	[80]	LIME, Counterfactual	Psychiatry	Ensemble	Helped predict therapy results.
36	2023	[81]	SHAP	Cardiology	RF	Atrial risk explanations.
37	2020	[82]	Attention, LIME	ICU/sepsis	RNN + GB	Temporal feature change.
38	2024	[83]	Grad-CAM	Dermatology	CNN	Psoriasis map validation.
39	2024	[84]	SHAP, IG	Oncology	Ensemble DL	Boosted oncologist trust.
40	2023	[85]	Attention	CDSS	Transformer	Explained medical dialogs.
41	2020	[86]	SHAP	CDSS	XGBoost	Ranked multi-risk features.
42	2022	[87]	Grad-CAM, attention	Ophthalmology	CNN + LSTM	Explained retina layers.
43	2025	[88]	LIME, SHAP	Psychiatry	RF	Interpreted anxiety scores.
44	2023	[89]	Attention, counterfactual	Oncology	Transformer	Simulated tumor changes.
45	2022	[90]	SHAP	Cardiology	Ensemble	Showed HF risk factors.
46	2020	[91]	Grad-CAM	Radiology	CNN	Identified pneumonia zones.
47	2021	[92]	SHAP, IG	Endocrinology	DNN	Consistent diabetes insight.
48	2022	[93]	Concept bottleneck, LIME	Pathology	Hybrid CNN	Linked visual concepts.
49	2023	[71]	SHAP, LIME	Neurology	XGBoost	Alzheimer’s risk profiles.
50	2020	[94]	LRP	Cardiology	CNN	Explained ECG rhythms.
51	2024	[95]	SHAP, Grad-CAM	Oncology	CNN + RF	Interpretable cancer model.
52	2023	[96]	Attention, IG	Neurology	Transformer	Seizure timing insights.
53	2023	[97]	SHAP, counterfactual	Psychiatry	XGBoost	Explained PTSD risk.
54	2022	[98]	Grad-CAM	Dermatology	CNN	CAM confirmed visually.
55	2024	[99]	LIME, IG	Cardiology	DNN	Identified heart failure signs.
56	2024	[60]	Concept bottleneck	Pathology	CNN	Linked histological features.
57	2025	[100]	SHAP, LIME	ICU care	XGBoost	Dual ICU explanation.
58	2022	[101]	Attention	Neurology	RNN	Highlighted EEG spikes.
59	2021	[102]	Grad-CAM	Radiology	CNN	COVID-19 CXR maps.
60	2023	[103]	SHAP	Oncology	RF	Explained gene profiles.
61	2024	[104]	IG, LIME	CDSS	Ensemble	Improved acceptance.
62	2025	[105]	SHAP, Grad-CAM	Multimodal CDSS	CNN + XGBoost	Integrated XAI framework.

**Table 5:** Frequency and use cases of XAI techniques. This table highlights how often each explainable AI method was used and summarizes their common applications in clinical decision support systems.

XAI Technique	Frequency	Common Use Case
SHAP	22	Feature attribution for tabular clinical data; prognosis, ICU risk scores, chronic condition monitoring
LIME	18	Local surrogate modeling; diagnostic rule generation, risk stratification
Grad-CAM	15	Visual explanations in CNNs for radiological images, pathology slides, dermatology scans
Attention mechanisms	9	Sequential data interpretation in time-series modeling, medical records, EEG/ECG data
Counterfactual explanations	6	Simulation of alternative scenarios for personalized treatment recommendations
Concept-based explanations	4	Bridging machine features with clinical semantics in explainable imaging models
Others (IG, LRP, DeepLIFT)	7	Gradient-based saliency for DL models in image and text interpretation

**Table 6:** Distribution of studies by clinical domain. This table presents the frequency of reviewed studies across medical specialties, along with representative clinical applications.

Clinical Domain	Frequency	Example Applications
Radiology	14	COVID-19 detection, pneumonia triage, lung segmentation
Oncology	13	Breast cancer prognosis, tumor classification, chemotherapy planning
Neurology	9	Seizure prediction, Alzheimer’s risk, EEG anomaly detection
Cardiology	8	Atrial fibrillation detection, heart failure risk, ECG analysis
ICU/critical care	6	Sepsis onset prediction, ICU mortality estimation
Endocrinology	4	Diabetes risk prediction, thyroid disease modeling
Dermatology	4	Skin cancer classification, lesion detection
Psychiatry	4	Depression risk modeling, PTSD analysis
Pathology	4	Histopathological image interpretation, biopsy feature saliency
Others (ophthalmology, primary care)	6	Retinal disease classification, multi-disease prediction

**Table 7:** Clinical usability strategies in the reviewed studies. This table summarizes the methods used to evaluate and enhance the practical adoption of XAI-CDSS tools in clinical settings.

Strategy	Count	Description
Clinician feedback	12	Expert reviews of explanations to determine clinical relevance, usability, and clarity
Human-in-the-loop trials	6	Real-time clinical task simulations with XAI-CDSS interaction
Prototype deployment	3	Integration of XAI models into clinical dashboards or EHR pilot testing
Trust/cognitive load surveys	5	Standardized questionnaires measuring trust, interpretability, and ease of use

**Table 8:** Prevalence of XAI methods by clinical data type. The table categorizes XAI methods based on their dominant application across imaging, structured data, and text/genomics modalities.

XAI Method	Imaging	Structured Data	Text/Genomics
SHAP	6	25	3
Grad-CAM	22	3	2
LIME	4	14	2
Attention mechanisms	2	2	14
Counterfactuals	1	2	2
Concept bottlenecks	0	1	2

**Table 9:** Stakeholder engagement and evaluation strategy in the reviewed studies.

Criterion	Count (%)
Clinician involvement in design/evaluation	18 (29%)
User trust scoring or perception studies	14 (22.5%)
Use of quantitative interpretability metrics (e.g., fidelity and sparsity)	26 (42%)
Open-source code or model release	18 (29%)
Reproducibility testing (cross-site or cross-data)	9 (14.5%)
Fairness/bias analysis or mitigation	6 (9.7%)

**Table 10:** TMEA design checklist linking clinical context to explanation choice and required evaluation.

Clinical Task	Modality	Candidate XAI Class	Primary Explanation Goal	Required Technical Metrics	Human Factors Metrics
Risk stratification	Tabular EHR	Model-agnostic attributions	Actionable factors	Fidelity, stability, monotonicity	Trust calibration, workload, time-on-task
Lesion localization	Imaging	Grad-CAM/CAM (model-specific)	Spatial verification	Localization IoU, pointing game, sanity checks	Error interception, overreliance reduction
Monitoring/ triage	Waveform/ time-series	Attention rationales, counterfactual timelines	Temporal reasoning	Consistency under perturbations, counterfactual validity	Timeliness, alarm fatigue, decision latency
Text classification	Clinical notes	Rationale extraction + counterfactuals	Evidence linking	Faithfulness, comprehensiveness/sufficiency	Explanation satisfaction, review time
Any high-risk task	Any	Hybrid (attribution + counterfactual)	Robustness/ verification	Stability–faithfulness trade-off, runtime	Escalation behavior, second-opinion seeking

**Table 11:** Research priorities and implementation strategies for future XAI in CDSSs. This table outlines key future directions and actionable strategies to enhance clinical integration and trust in explainable AI systems.

Future Research Area	Implementation Strategy
Standard metrics	Develop consensus benchmarks, use domain expert panels, validate against clinical decisions
Stakeholder design	Conduct multi-user workshops, feedback loops in development cycles
Clinical validation	Longitudinal studies, registry-based trials, multi-center reproducibility checks
Ethical alignment	Implement bias audits, transparent reporting checklists, regulator involvement
Multimodal XAI	Cross-domain training pipelines, explainers fused with multimodal inputs
AI education	CME-accredited programs, interactive web tools, simulated case evaluations
Human–AI teaming	Personalized UIs, context-aware explainer APIs, voice-interactive modules

**Table 12:** Summary of the study limitations and their implications. This table outlines the methodological and scope-related limitations in the review, along with their potential effects on the findings and generalizability.

Limitation	Potential Impact
Language and publication bias	Exclusion of non-English and non-indexed studies may skew conclusions
Inconsistent reporting	Reduces reproducibility and cross-study comparability
Qualitative synthesis subjectivity	Reviewer interpretation may affect categorization and trend identification
Rapid evolution of XAI	Some recent methods not reflected in dataset due to publication lag
No meta-analysis	Limits the statistical strength of synthesis and risk of bias estimation
Domain skewness	May not reflect modality-specific requirements for underrepresented data types
Lack of diverse stakeholders	Omits critical perspectives from patients and non-clinical users

## Data Availability

No new data were created or analyzed in this study.
